# Audio-Tactile Skinny Buttons for Touch User Interfaces

**DOI:** 10.1038/s41598-019-49640-w

**Published:** 2019-09-16

**Authors:** Quang Van Duong, Vinh Phu Nguyen, Anh Tuan Luu, Seung Tae Choi

**Affiliations:** School of Mechanical Engineering, Chung-Ang University, 84 Heukseok-Ro, Dongjak-Gu, Seoul 06974 Republic of Korea

**Keywords:** Actuators, Polymers

## Abstract

This study proposes a novel skinny button with multimodal audio and haptic feedback to enhance the touch user interface of electronic devices. The active material in the film-type actuator is relaxor ferroelectric polymer (RFP) poly(vinylidene fluoride-trifluoroethylene-chlorofluoroethylene) [P(VDF-TrFE-CFE)] blended with poly(vinylidene fluoride-trifluoroethylene) [P(VDF-TrFE)], which produces mechanical vibrations via the fretting vibration phenomenon. Normal pressure applied by a human fingertip on the film-type skinny button mechanically activates the locally concentrated electric field under the contact area, thereby producing a large electrostrictive strain in the blended RFP film. Multimodal audio and haptic feedback is obtained by simultaneously applying various electric signals to the pairs of ribbon-shaped top and bottom electrodes. The fretting vibration provides tactile feedback at frequencies of 50–300 Hz and audible sounds at higher frequencies of 500 Hz to 1 kHz through a simple on-off mechanism. The advantage of the proposed audio-tactile skinny button is that it restores the “click” sensation to the popular virtual touch buttons employed in contemporary electronic devices.

## Introduction

Multimodal human-machine interfaces are becoming increasingly popular in a wide range of applications for enhancing the quality of human-machine interaction and the user experience when users scan or touch the machine surface with their fingertips^1–6^. Notably, mechanical button, one of the simplest user interfaces (UIs), can even provide multimodality in the form of simultaneous kinesthetic force, tactile, and aural feedback, resulting in a click sensation for users. However, mechanical buttons have been increasingly replaced with touch buttons, including capacitive^7–12^, resistive^13,14^ and inductive^15–17^ touch buttons, in which the kinesthetic force feedback is removed for aesthetic reasons. The “click” sensation that occurs at the state-transition moment of a mechanical button can be restored using artificial beep sounds or vibration feedback. While sound feedback is relatively simple, vibration feedback remains unsatisfactory. Conventional gross mode actuators such as the eccentric motor^18,19^, piezoelectric actuator^20–22^, and linear resonator^23,24^ cannot effectively provide localized vibrations for multiple touch buttons. Virtual buttons on touchscreens can be combined with tactile feedback through, electrovibration^25–30^ or ultrasonic vibration^31^, for example. However, such friction-based vibrations require the movement of the users’ fingertip. Therefore, the development of tactile (vibration) feedback for touch buttons under static pressure that mimics the “click” sensation of mechanical buttons is an ongoing research challenge.

In this study, a novel audio-tactile skinny touch button is proposed that provides both tactile and aural sensations via the fretting vibration phenomenon of blended relaxor ferroelectric polymer (RFP) films. The audio-tactile skinny touch button is composed of a flexible touch layer, a spacer, and a substrate. The flexible touch layer has a scratch-resistant cover film, a touch sensor, a top electrode, and an RFP film. A bottom electrode is placed on top of the substrate, and the spacer layer separates the RFP film and the bottom electrode with a uniform air gap. The top and bottom electrodes are patterned to ensure that they have the same ribbon-shaped active areas so that various electrical signals can be applied simultaneously to top and bottom electrode pairs. When a human fingertip applies normal pressure to the touch button, a local electric field is mechanically activated on the contact area between the RFP film and the bottom electrode, which produces a large electrostrictive strain in the RFP film. The synergetic interplay among the localized electric field, electrostrictive deformation of the RFP film, and contact area dramatically amplifies the vibration of the flexible touch layer. The concept of vibration amplification in RFP film via the fretting vibration phenomenon was first introduced by Duong *et al*.^32^ for localized vibrotactile sensation in large-area displays. In this study, the same vibration amplification mechanism is applied for tactile vibration as well as for audible sound generation with ribbon-shaped electrodes. When electrical voltage signals with frequencies ranging from 50 Hz to 300 Hz or 500 Hz to 20 kHz are applied to the ribbon-shaped electrodes of one skinny touch button, tactile vibrations or audible sound are simultaneously generated, respectively. Poly(vinylidene fluoride-trifluoroethylene-chlorofluoroethylene) [P(VDF-TrFE-CFE)] and poly(vinylidene fluoride-trifluoroethylene-chlorotrifluoroethylene) [P(VDF-TrFE-CTFE)] are well-known RFPs^33^. RFPs produce large electrostrictive strain of up to approximately −7% under an electric field of 150 V/µm^34,35^. In order to produce enhanced electrostrictive strain, P(VDF-TrFE-CFE) (80 wt%) blended with P(VDF-TrFE) (VDF:TrFE = 55:45 by molar fraction) (20 wt%) is used as the RFP^32,36^. The skinny button provides the tactile-feedback vibrations at frequencies of 50 Hz to 300 Hz and audible sounds at higher frequencies of 500 Hz to 20 kHz via a simple on-off mechanism. With this simple structure, the audio-tactile skinny button can be integrated with capacitive touch sensors, resistive touch sensors or inductive touch sensors without any physical interference. The audio-tactile skinny button developed in this study has the ability to provide the multimodal “click” sensation currently lacking from touch buttons in electronic devices.

## Results and Discussion

### Functional mechanism of the audio-tactile skinny button

**Figure 1:**
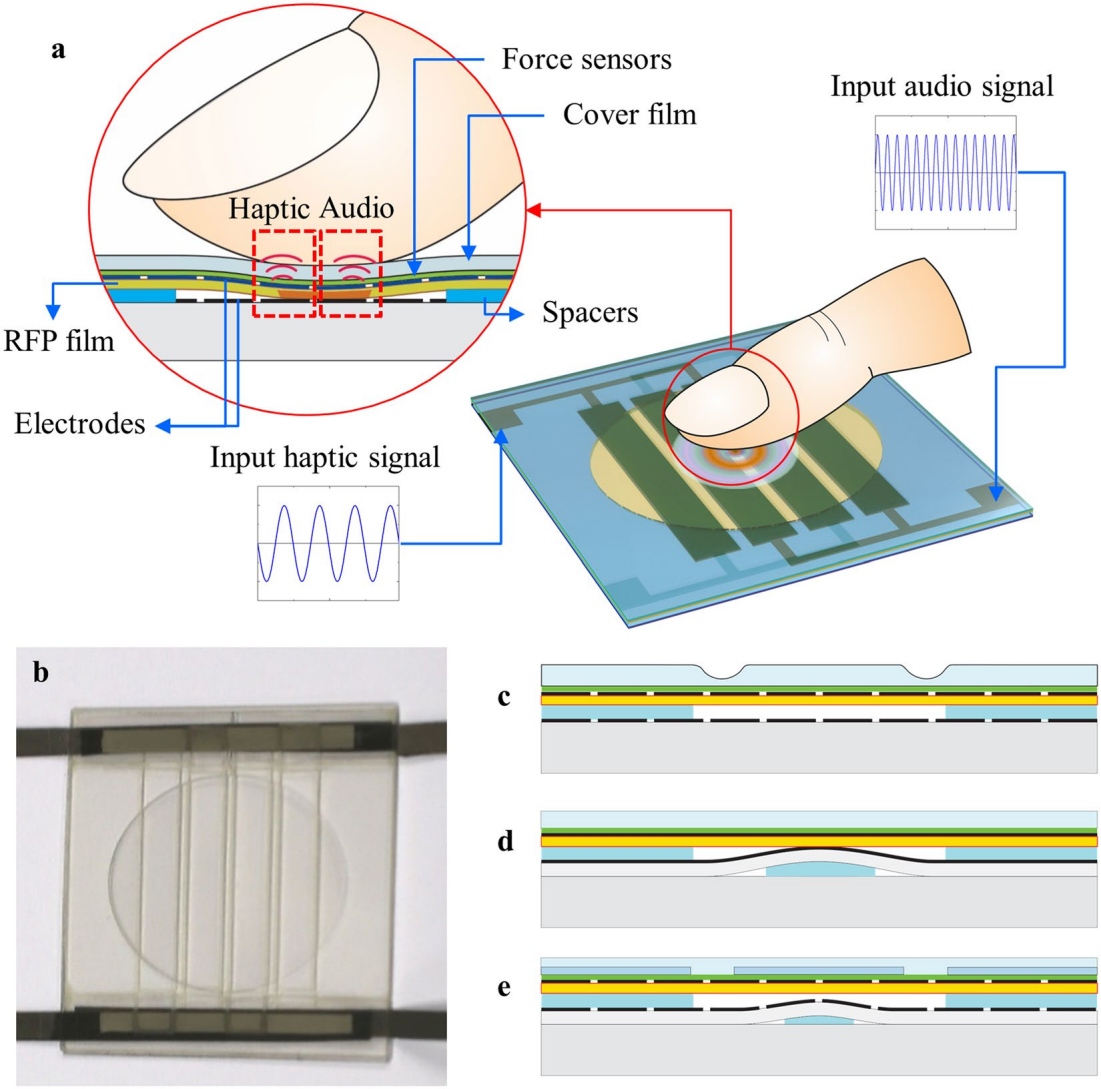
Design and functional mechanism of the audio-tactile skinny button. (**a**) Conceptual design and functional mechanism of the flexible audio-tactile skinny button, in which aural and haptic sensations are produced simultaneously via the fretting vibration phenomenon. **(b)** Photograph of the audio-tactile skinny button fabricated in this study. **(c**–**e)** Different designs of the audio-tactile skinny button: **(c)** with a circumferential dimple on the flexible cover layer, which reduces the force required to activate the button; **(d)** with a bump at the center, which generates the haptic vibration without any fingertip contact; and **(e)** with a bump at the center, which reduces the force required force to activate the button.

Figure 1a shows the conceptual design and functional mechanism of the audio-tactile skinny button developed in this study, which provides tactile feedback as well as aural feedback. The audio-tactile skinny button consists of three parts: a flexible touch layer, a spacer, and a substrate. The flexible touch layer comprises a touch sensor located underneath a scratch-resistant polyethylene terephthalate (PET) cover, a top electrode made of silver nanowires (AgNWs), and a blended RFP film. The bottom AgNW electrode is deposited on the substrate. The flexible touch layer and substrate are attached with the spacer between them to maintain a uniform gap. The top and bottom electrodes are patterned to create an array of ribbon shape actuators as shown in Fig. 1a. Therefore, various voltage signals with different amplitudes, profiles, and frequencies can be applied to the various pairs of electrodes to simultaneously provide the tactile vibration and audible sound. When a fingertip applies a normal pressure to the flexible touch cover, resulting in contact between the RFE film and bottom electrodes, concentrated electric fields are mechanically activated under the contact area. The high electric field, electrostrictive deformation of the RFP film, and contact area combine to amplify the vibrations for tactile sensation and audio recognition. Figure 1b shows the audio-tactile skinny button fabricated in this study (refer to Supplementary S1 for the detailed fabrication process). Figure 1c–e demonstrates the various designs of the audio-tactile skinny button. In order to reduce the force required to activate the fretting vibration, the flexible touch layer can be equipped with a circumferential dimple as shown in Fig. 1c. If a bump is located underneath the bottom electrode and permanent contact is achieved between the RFP film and bottom electrode as shown in Fig. 1d, a human fingertip can feel the vibration with a gentle touch on the button. Figure 1e represents another variation of the audio-tactile skinny button, in which a stiff membrane reinforces in the flexible cover layer and a bump underneath the bottom electrode adjusts the stiffness of the flexible cover layer and the force required to activate the fretting vibration.

### Finite element analysis and experimental measurement of fretting vibration

**Figure 2:**
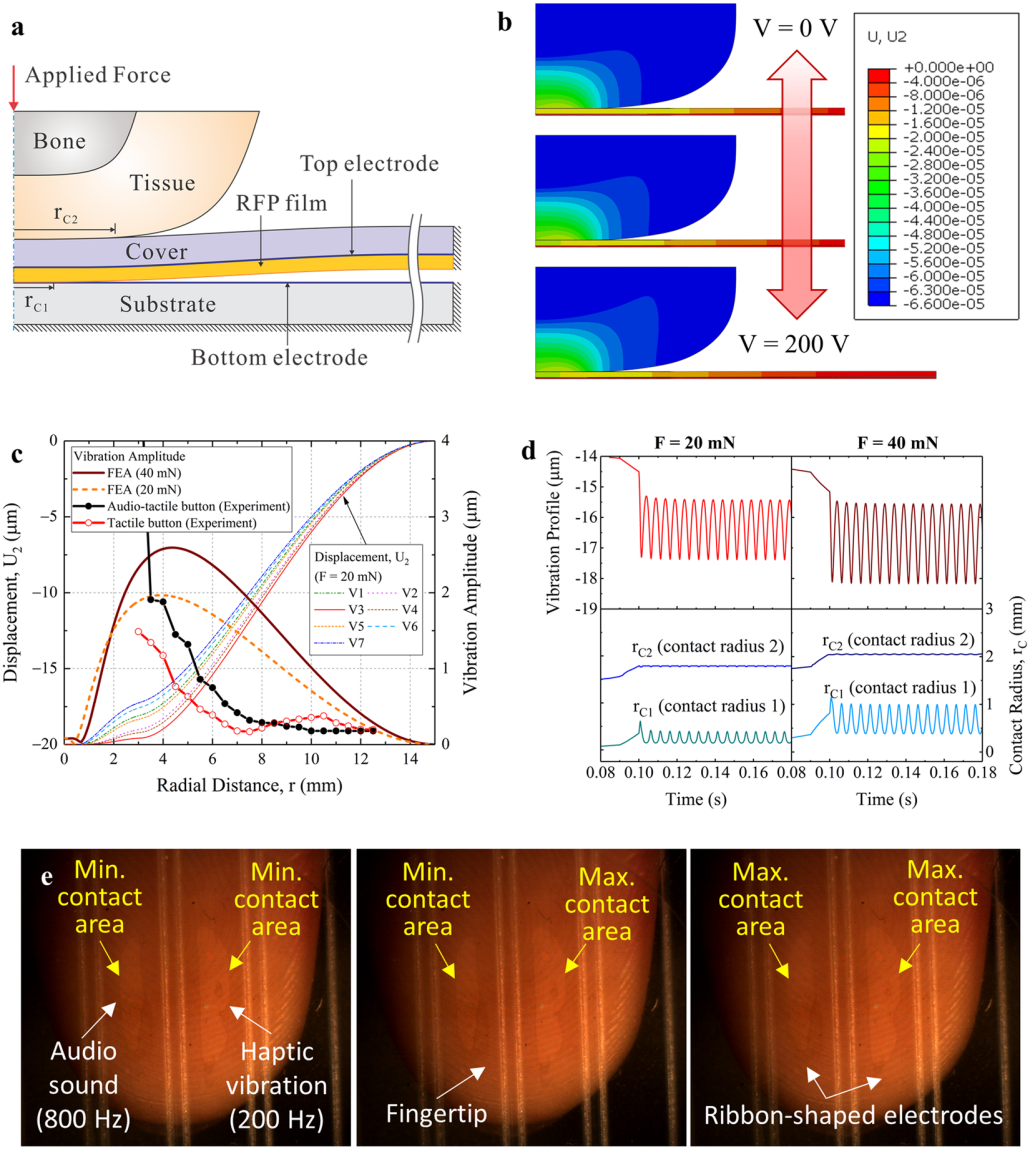
FEA and experimental measurement of vibrations in audio-tactile skinny buttons. (**a)** Axisymmetric finite element model of the button composed of a PET cover, RFP film, glass substrate, and fingertip (including bone and tissue parts). **(b)** FEA snapshots of deformed shapes and displacement fields of the fingertip and flexible touch layer at various applied voltages. **(c)** Displacement profiles (FEA results) and vibration amplitudes (experimental and FEA results) along the radial distance from the center of the flexible touch layer. **(d)** FEA results of vibration profiles at r (radial distance) = 4.6 mm and contact radii (contact radius 1 and contact radius 2) as a function of time. **(e)** Snapshots of the contact areas between the RFP film and bottom electrodes measured with a high-speed camera.

To examine the functional mechanism via the fretting vibration phenomenon, the haptic button plus fingertip shown in Fig. 2a is modeled and evaluated through axisymmetric dynamic finite element analysis (FEA). A sinusoidal voltage $V(t)={V}_{max}(1+\,\sin \,2\pi \omega t)/2$ with *V*_*max*_ = 200 V and *ω* = 200 Hz is applied through the top and bottom electrodes when contact is achieved between the RFP film and the bottom electrode under applied pressure of the fingertip (Fig. 2a). Here, *r*_*C*1_ represents the contact radius between the bottom electrode and the RFP film, and *r*_*C*2_ is the contact radius between the flexible cover layer and the fingertip. Figure 2b shows the contour plots of the typical vertical displacement during one cycle of applied voltage, which demonstrates the vibration, i.e., the upward and downward movement of the flexible cover layer. To analyze the vibration profile and vibration amplitude of the device in more detail, two cases of static force applied through a human fingertip are considered (*F* = 20 mN and 40 mN). The applied force is increased in the first 0.1 s to the desired value of 20 mN or 40 mN and then kept constant throughout the simulation. Figure 2c plots the vertical displacement profiles and vibration amplitudes as a function of the radial distance from the contact center. The vibration amplitude in the area under contact with the fingertip (radial distance ≤ 1 mm) corresponds to the electrostrictive strain of the RFP film. Outside the contact area (radial distance > 1 mm), the vibration amplitudes increase until the maximum value at radial distance of approximately 4 mm and then gradually decrease. In the case of *F* = 20 mN, the maximum vibration amplitude is 1.97 µm with *r*_*C*2_ = 1.81 mm and *r*_*C*1_ = 0.31 mm (Fig. 2d). When the applied force is 40 mN, the maximum vibration amplitude is 2.59 µm with *r*_*C*2_ = 2.06 mm and *r*_*C*1_ = 0.69 mm (Fig. 2d). Therefore, by varying the applied force through the fingertip, i.e., pressing more lightly or more firmly, the user can perceive different levels of tactile sensation from the skinny button.

In addition to the FEA, the vibration amplitudes of the audio-tactile skinny button with patterned electrodes and the tactile button with uniform electrodes were also measured by a laser displacement sensor (LDS) (refer to the Methods). Polydimethylsiloxane (PDMS), which has mechanical properties similar to a human fingertip, is utilized to fabricate a bump with a radius of 3.0 mm for applying static pressure on the flexible touch layer. The measured vibration amplitude of the flexible cover layer as a function of the radial distance is plotted together with the vibration amplitude obtained from FEA in Fig. 2c. For the audio-tactile skinny button, the maximum vibration amplitude is obtained near the contact area, and the amplitude decreases from 5.40 µm to 0.18 µm as the radial distance increases from 3.0 mm to 12.5 mm. A similar trend is observed for the tactile button but with a smaller vibration amplitude. A more detailed analysis and discussion is provided in the next section on the fretting-vibrotactile sensation. It is worth noting that the audio-tactile skinny button and the axisymmetric model in FEA have different operational conditions. The audio-tactile skinny button has top and bottom electrodes patterned into ribbon-shaped areas and therefore ribbon-shaped active areas in the RFP actuator, whereas the RFP film and top and bottom electrodes in the axisymmetric finite element model are uniform. However, the experimental results of vibration amplitude as a function of radial distance are generally in agreement with the FEA results, as the underlying mechanism in both cases is still the fretting vibration phenomenon. Figure 2e shows snapshots of the contact areas between RFP film and bottom electrodes measured with a high-speed camera (refer to Supplementary S2 for the measurement technique and the Supplementary S3 for the full movie). When two voltage signals with different frequencies (200 Hz and 800 Hz) are applied to the ribbon-shaped electrode pairs, the contact areas vibrate with different frequencies.

### Fretting-vibrotactile sensation from the audio-tactile skinny UI button

**Figure 3:**
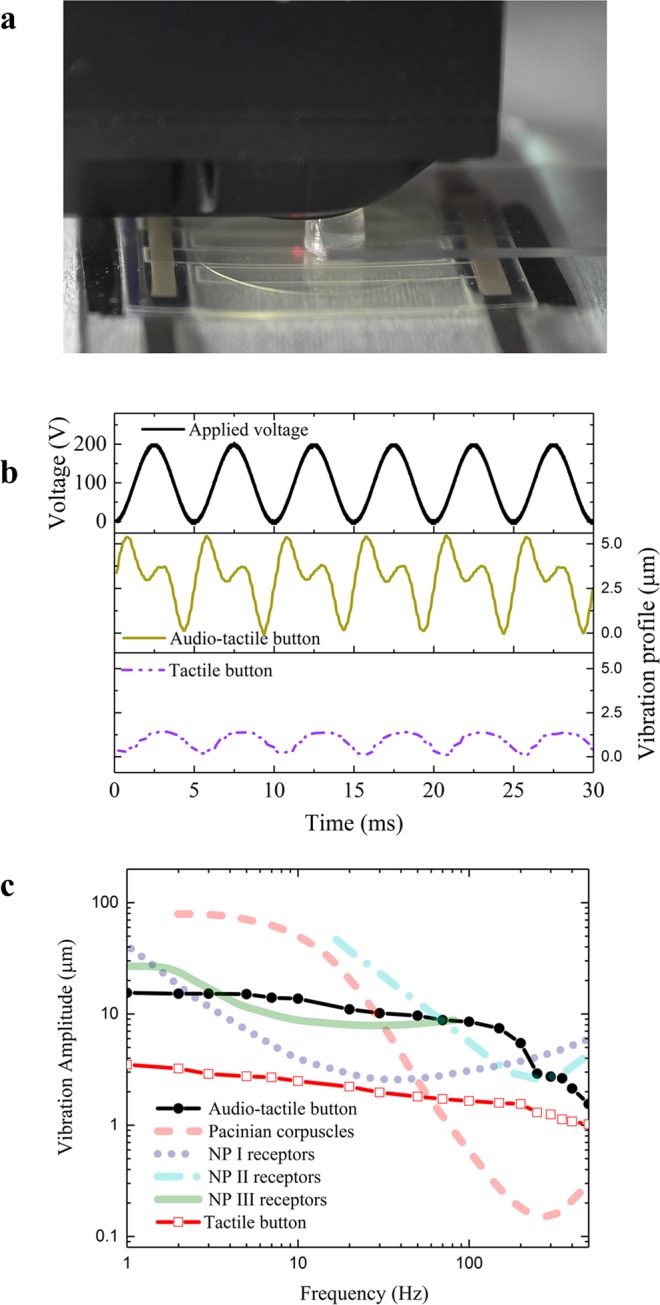
(**a**) Experimental set-up of the fretting vibration measurement of the skinny button. **(b)** Applied sinusoidal voltage of *V*_*max*_ = 200 V and vibration profiles. **(c)** Vibration amplitude as a function of frequency, when the sinusoidal voltage given in **(b)** is applied^37–39^.

An audio-tactile skinny button is fabricated following Supplementary S1 and shown in Fig. 1b. Notably, the audio-tactile skinny button simultaneously provides the audio and haptic feedback via a simple on-off mechanism through normal pressure of the human fingertip. The haptic feedback induced by fretting vibration is discussed here and audio feedback is examined in the following section. As shown in Fig. 3a, a PDMS bump is used to apply the static force on the flexible cover of the skinny button. A sinusoidal voltage with *V*_*max*_ = 200 V is applied between the top and bottom electrodes and the vibration amplitude near the contact area on the top of the flexible cover is measured with an LDS. Figure 3b shows the vibration profile of the audio-tactile skinny button and the tactile button at 200 Hz with the vibration amplitude about 5.4 µm and 1.2 µm, respectively. Interestingly, the vibration profiles of those two button types exhibit important differences. The audio-tactile skinny button produces larger vibration amplitudes and its vibration profile in one period seems to have an additional small peak. This may be due to the differences in the localized electric field and RFP film active areas under the fretting vibration phenomenon, which varies the resistance of the flexible touch layer to vibration. For the haptic button, the localized electric field, electrostrictive strain, and vibration are axisymmetric, so the whole cover has to be stretched and oscillated in every direction from the contact area, resulting in greater resistance and smaller vibration amplitude. Also, the axisymmetric properties of the haptic button produce a smoother vibration profile, whereas the asymmetric characteristics of the audio-tactile skinny button make the vibration profile large and complicated due to the electric field variations and resulting variability in fretting vibration directions. As the frequency of vibration plays an important role in tactile sensation, it is important to determine how the vibration of the skinny button depends on the applied frequency. Specifically, the vibration amplitude affects a human’s tactile sensation threshold. The change in vibration amplitude of the audio-tactile skinny button as a function of frequency is shown in Fig. 3c; it reaches almost 15.5 µm at 1 Hz. As the operating frequency increases, it gradually decreases to approximately 1.6 µm at 500 Hz. The tactile button exhibits the same trends at lower values of vibration amplitude.

In order to explain how the fretting vibrations from the skinny button can provide haptic feedback to a human finger, it is crucial to understand the sensory receptors of the human fingertip. Human fingertips can detect mechanical stimuli through four mechanoreceptors under the skin^37–39^, which are then divided into two main groups: slow-adapting (SA) receptors (i.e., NP II (Ruffini corpuscles) and NP III (Merkel cells)^39–41^) and fast-adapting (FA) receptors (i.e., Pacinian corpuscles (PC) and Meissner corpuscles or NP I (non-Pacinian)). The SA receptors are sensitive to the pressure, texture, and form of an object and can detect the threshold at low frequencies (0.4–10 Hz). At intermediate frequencies between 1.5 Hz and 50 Hz, the threshold is detected by the NP I channel. The PCs respond to stimuli at high frequency (50–300 Hz) and are highly sensitive to changes in the stimulus size, skin-surface temperature, and duration^38,39,42–44^. When the user presses and applies a force on the flexible touch layer of the skinny button, the NP III in the user’s fingertip is immediately stimulated. Note that, among SA receptors, the NP III is sensitive to indentations and normal pressures, whereas NP II responds to skin stretching^45,46^. When fretting vibration occurs, the stimulus depends on the vibration amplitude and frequency in relation to the thresholds of all mechanoreceptors, which are plotted in Fig. 3c. Interestingly, the skinny button fires the Meissner corpuscles (NP I) and Merkel cells (NP III) at low frequencies of 5–20 Hz; therefore, it can be used to mimic the texture or form of an object when operating at low frequency. From 30–80 Hz, most of the mechanoreceptors (NP I, NP III, and PC) are stimulated. At higher frequencies up to 500 Hz, the Pacinian corpuscles are most sensitive to fretting vibration from the skinny button. The minimum threshold amplitude of Pacinian corpuscles is approximately 0.2 µm at close to 200 Hz, which is the minimum threshold amplitude for the haptic sensation of human fingertips. Therefore, the measured vibration amplitude of the audio-tactile skinny button is sufficiently large to be perceived by a human fingertip, and the applied voltage (frequency, amplitude) can be controlled to selectively stimulate mechanoreceptors for various haptic stimuli.

### Audio feedback from the audio-tactile skinny UI button

**Figure 4:**
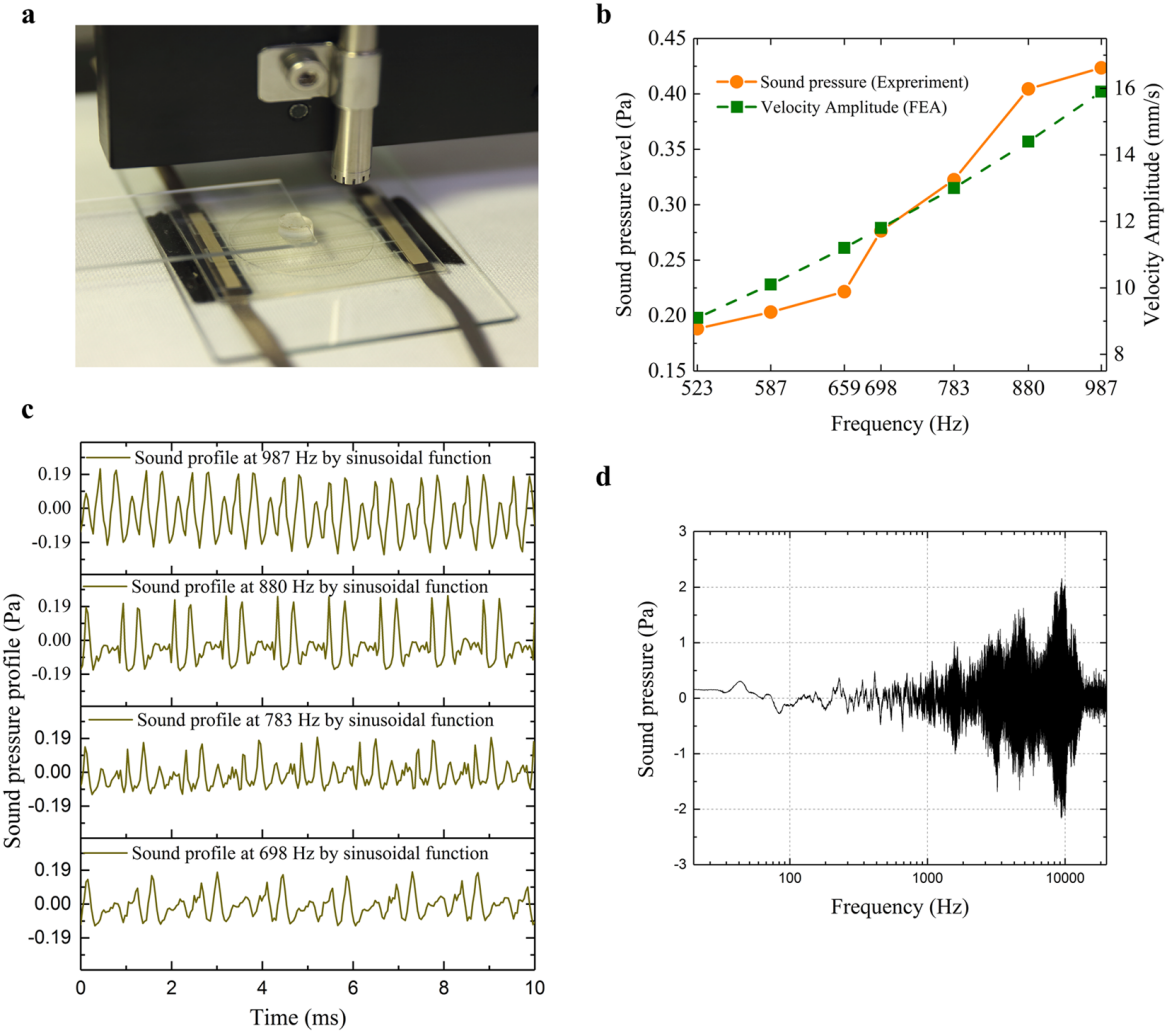
(**a**) Experimental set-up of the sound measurement of the skinny button. **(b)** Velocity amplitude (FEA results) of the flexible touch layer of the audio-tactile button under a sinusoidal voltage of  *V*_*max *_ = 400 V and corresponding sound pressure level (experimental results). **(c**) Sound pressure profile of the audio-tactile button with different frequencies. **(d)** Measured sound pressure of the audio-tactile skinny button as a function of frequency.

The detailed analysis of the sound feedback of the skinny button is discussed in this section (Fig. 4). From a physical perspective, sound is a pressure wave typically created by vibration of an object. The perception of sound by the human ear is affected by several characteristics of the received sound waves such as intensity and frequency. In the case of our skinny button, the acoustic vibrations generated by the flexible touch layer are transformed into acoustic waves that travel through the air; the intensities of these waves give the sound pressure. Wave intensity is measured by applying a sinusoidal voltage of *V*_*max*_ = 400 V to the top and bottom electrodes and recording the sound pressure with a microphone (Fig. 4a). The results in the frequency range of 523–987 Hz, which is equivalent to one octave scale, are shown in Fig. 4b.

The sound pressure level reaches 0.18 Pa at 523 Hz and increases monotonically with frequency up to 0.42 Pa at 987 Hz. Note that it is measured approximately 1 cm from the skinny button and should be inversely proportional to the distance of sound reception due to the nature of spherical sound wave propagation^47^. As for the human sensation of sound, the distance from the button to the human ear (approximately 1 m) should be considered; thus, the effective sound pressure level for hearing in normal situations ranges from approximately 1.8 mPa at 523 Hz to 4.2 mPa at 987 Hz, which is above the auditory threshold of the human ear (0.02 mPa)^48^. FEA of the skinny button with a fingertip (Fig. 2a) is performed for comparison and the velocity of the flexible touch layer is plotted in Fig. 4b. In theory, the velocity and sound pressure are proportional^47^; therefore, the FEA results are in general agreement with the measured sound pressure level. The detailed sound pressure profiles for some frequencies, which are important for more subtle attributes of sound such as pitch and timbre, are shown in Fig. 4c. The different profiles are obtained by applying different electric signals; therefore, the skinny button can be controlled to produce different tones. Finally, the frequency response in the entire audible range of the human ear (20 Hz – 20 kHz) is measured in Fig. 4d. The skinny button presented here generates audible sound from 250 Hz to 20 kHz with a strong sound pressure level in the frequency range of 1–10 kHz.

### Demonstration of various audio-tactile skinny buttons

**Figure 5:**
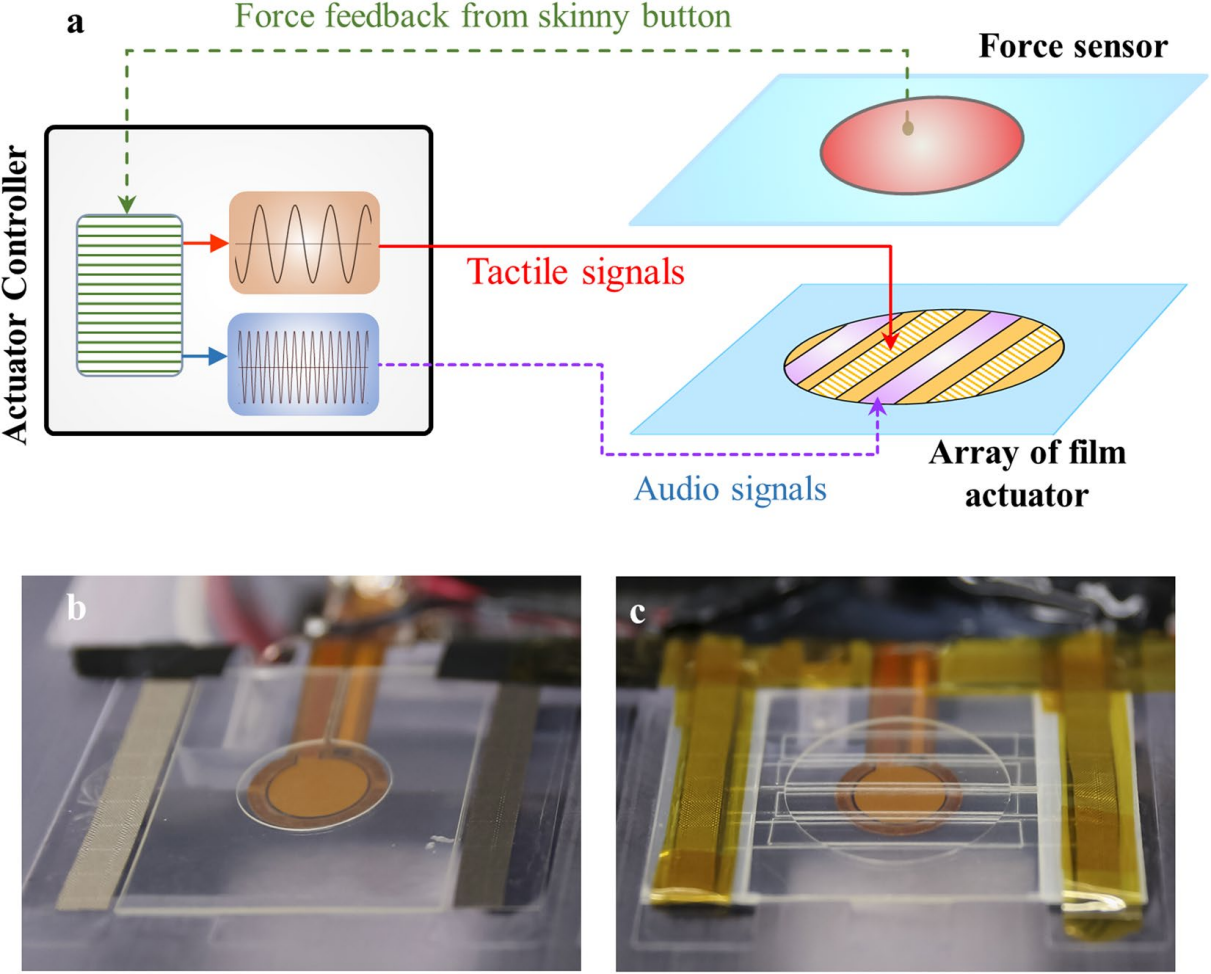
Demonstration and functional mechanism of the tactile skinny buttons. (**a**) Schematic diagram of the control algorithm for the audio-tactile skinny buttons. Demonstration of (**b)** the pressure-responsive tactile skinny button and **(c)** the pressure-responsive audio-tactile skinny button.

Figure 5a represents a schematic diagram of the control algorithm for an audio-tactile interface button, and Fig. 5b,c demonstrate the pressure-responsive tactile skinny button and audio-tactile skinny button, respectively, integrated with a force sensor to provide the users’ touch input for the controller. The electrodes are patterned into an array of ribbon-shaped areas in order to activate and control different parts of the RFP film, which are equivalent to an array of ribbon actuators. When a human fingertip provides static pressure on the flexible touch layer of the button, the actuator controller detects the force from the fingertip through the force sensor, then sends various electric signals to the functional ribbon actuators. The ribbon actuators generate tactile feedback and audio feedback depending on the operation frequencies via a simple on-off mechanism activated by a human fingertip. Here, the simple on-off mechanism activates the fretting vibration when there is contact between the RFP film and the bottom electrode under applied pressure, whereas the controller detects the user’s touch before contact with the RFP film and introduces appropriate electric signals to produce the desired feedbacks. It should be noted that the capacitive touch sensors can also be integrated in the button instead of the force sensors.

## Conclusion

In this study, a multimodal audio-tactile skinny button based on the fretting vibration phenomenon in an RFP film was developed and presented. Pressure applied by a human fingertip activates a localized alternating electric field under the contact area, which produces a variation in the electrostrictive strain of the RFP film and generates an amplified mechanical vibration. The top and bottom electrodes are patterned into pairs of ribbon-shaped areas; thus, they effectively divide the RFP film into corresponding active areas that can operate simultaneously. In this manner, multimodal audio and tactile feedback can be achieved by applying multiple electric signals with appropriate frequencies. The results show that the skinny button can provide tactile sensations and audible sound at frequencies of 50–300 Hz and 0.5–20 kHz, respectively, thereby covering a wide range of possible stimuli for the human senses. Moreover, several designs of the skinny button are proposed, which enable adjustment of its mechanical properties such as stiffness and sensitivity to pressure. The skinny button presented in this study provides the desired “click” sensation through multimodal audio and haptic feedback, resulting in an enhanced user interface for virtual touch buttons in contemporary electronic devices.

## Methods

### Preparation of the top and bottom electrodes

0.1 wt% AgNWs measuring 25 µm long and 30 nm wide were used in deionized (DI) water as the flexible and transparent electrode. The AgNWs were deposited on the substrate by a solution casting method. Then, the AgNWs were dried in an oven at 85 °C for 30 min. The AgNW electrode was patterned into a ribbon shape using a CO_2_ laser.

### Preparation of the blended RFP films

The relaxor ferroelectric terpolymer VDF/TrFE/CFE (59.8/32.2/8 molar ratio) and the normal ferroelectric copolymer VDF/TrFE (55/45 molar ratio) were synthesized at Piezotech S.A. in France. 10 g of P(VDF-TrFE-CFE) terpolymer was dissolved in 90 g of methyl isobutyl ketone (MIBK), and 10 g of P(VDF-TrFE) copolymer was dissolved in 90 g of methyl ethyl ketone (MEK). Then, the terpolymer solution and the copolymer solution were mixed together. Blended RFP film with a thickness of 5 µm was fabricated with the RFP solution according to the adhesion-mediated film transfer (AMFT) technique^49^.

### Integration of the haptic skinny button

The RFP film was laminated onto a top electrode at 120 °C by the Excelam Plus 355R roll laminator. Optically clear adhesive film with a thickness of 50 µm was used as the spacer to connect the RFP film and bottom electrode

### Evaluation of the tactile feedback of the skinny button

The tactile feedback of the skinny button was evaluated by measuring the acoustic vibration on the top cover films with the LT-9010 (KEYENCE) LDS. The skinny button was placed on a vibration isolation table, and static pressure was applied on the device by a PDMS bump with a radius of 3.0 mm. A sinusoidal waveform voltage with a frequency of 200 Hz and *V*_*max*_ = 200 V was generated by an arbitrary waveform generator (Agilent, Model: 33210A) and high-voltage amplifier (MATSUSADA, Model: HEOPT-2B20–02). The tactile device was operated with the voltage signal, and its out-of-plane vibration was measured by the LDS placed on xyz-manual stages. The measured vibration and applied voltage signal were recorded with an oscilloscope.

### Evaluation of the audio feedback of the skinny button

The audio feedback of the skinny button was measured by the same method as that for evaluation of tactile feedback. In this case, the maximum sinusoidal waveform voltage was 400 V. The sound pressures of the skinny button were measured by a GRAS 40PH array microphone.

### Dynamic finite element analysis

FEA of the dynamic thermomechanical contact of the haptic button with a fingertip (Fig. 2a,b) was performed with ABAQUS^50^. An axisymmetric model of the haptic button (comprising a glass substrate, RFP film, and PET cover) and fingertip (comprising bone and tissue) was used to evaluate the fretting vibration. The mechanical responses of the RFP film were simulated thermomechanically via positive and negative thermal expansion phenomena, which are analogous to the electrostrictive effects and feasible for modeling with commercial software such as ABAQUS. Note that, for simplicity, the axisymmetric model was utilized for FEA of the fretting vibration phenomenon according to the individual frequency of the applied electric signal, which is useful for describing and explaining the general mechanical vibration trends.

## Supplementary information

Supplementary Information

Supplementary movie S3
