# Supplementary material for: Audio-Tactile Skinny Buttons for Touch User Interfaces
Source: Sci Rep. 2019 Sep 16;9:13290. doi: 10.1038/s41598-019-49640-w (PMC6746798; doi:10.1038/s41598-019-49640-w)
Supplement: Supplementary file 1 — Supplementary Information [file 41598_2019_49640_MOESM1_ESM.doc]

Supplementary Information

# **Audio-Tactile Skinny Buttons for Touch User Interfaces**

Quang Van DUONG, Vinh Phu NGUYEN, Anh Tuan LUU, and Seung Tae CHOI

*School of Mechanical Engineering, Chung-Ang University, 84 Heukseok-Ro, Dongjak-Gu, Seoul 06974, Republic of Korea*

Correspondence and requests for materials should be addressed to S.T.C. (email: stchoi@cau.ac.kr)

Table of Contents

S1. Fabrication Procedure of the Audio-Tactile Skinny Button

S2. Observation of the Fretting Vibration Phenomenon

S3. Full Movie of the Fretting Vibration Phenomenon

S1. Fabrication Procedure of the Audio-Tactile Skinny Button

The preparation procedure of the audio-tactile skinny button is illustrated in Fig. S1. The cross-sectional view and top view of the functional layer are shown in Fig. S1a and S1c. The fabrication process for the audio-tactile button consists of six main steps, as shown in Fig. S1b. The silver nanowires (AgNWs), which act as an excellent electrode with high electrical conductivity, flexibility, and high optical transparency that can be cured at low temperature^1-3^, are directly deposited on the force sensor for the top electrode (cover/force sensor/AgNWs). Then, the top electrode is patterned into the ribbon shape by a laser patterning technique. A highly transparent and flexible RFP film measuring 3–4 µm, which is fabricated and handled using an adhesion-mediated film transfer (AMFT) technique^4^, is laminated onto the top electrode at 120 °C to generate the film type actuator. Additionally, the AgNWs are coated and patterned on the substrate as the bottom electrode. Finally, the flexible touch layer, consisting of the cover/force sensor/top electrode/RFP film, is placed on the bottom part of the button, consisting of the bottom electrode/substrate, with a uniform gap formed by the spacers.


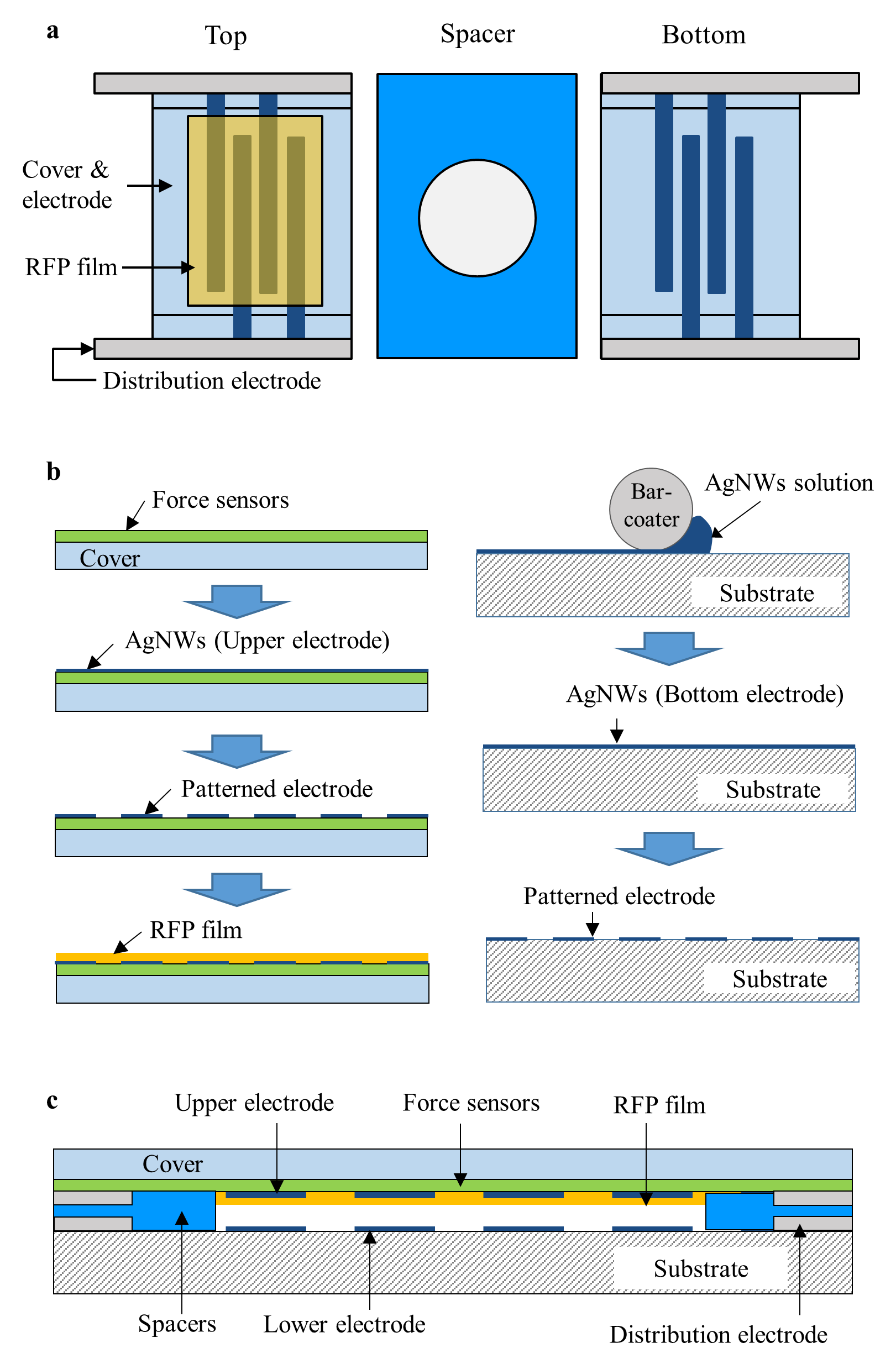


**Figure S1.** Schematic illustration of the fabrication steps for a flexible audio-tactile skinny button based on RFP film: **(a)** top view of the key components; **(b)** schematic fabrication process; and **(c)** cross-sectional view of the final audio-tactile skinny button.

S2. Observation of the Fretting Vibration Phenomenon

Figure S2 shows the experimental setup for observing the fretting vibration phenomenon. The audio-tactile skinny button is flipped and fixed onto a manual stage placed on a vibration isolation table. The Infiniprobe TS-160 is mounted on the commercial Photron FASTCAM Mini UX100 high-speed camera and placed on top of the audio-tactile skinny button. Sinusoidal voltages with a maximum of 200 V at 200 Hz and 400 V at 800 Hz are applied to various pairs of top and bottom electrodes. With a human fingertip, a force is applied to the flexible touch layer to make contact between the RFP film and bottom electrodes. A high-speed camera then captures the fretting vibration with a frame rate of 5000 fps.


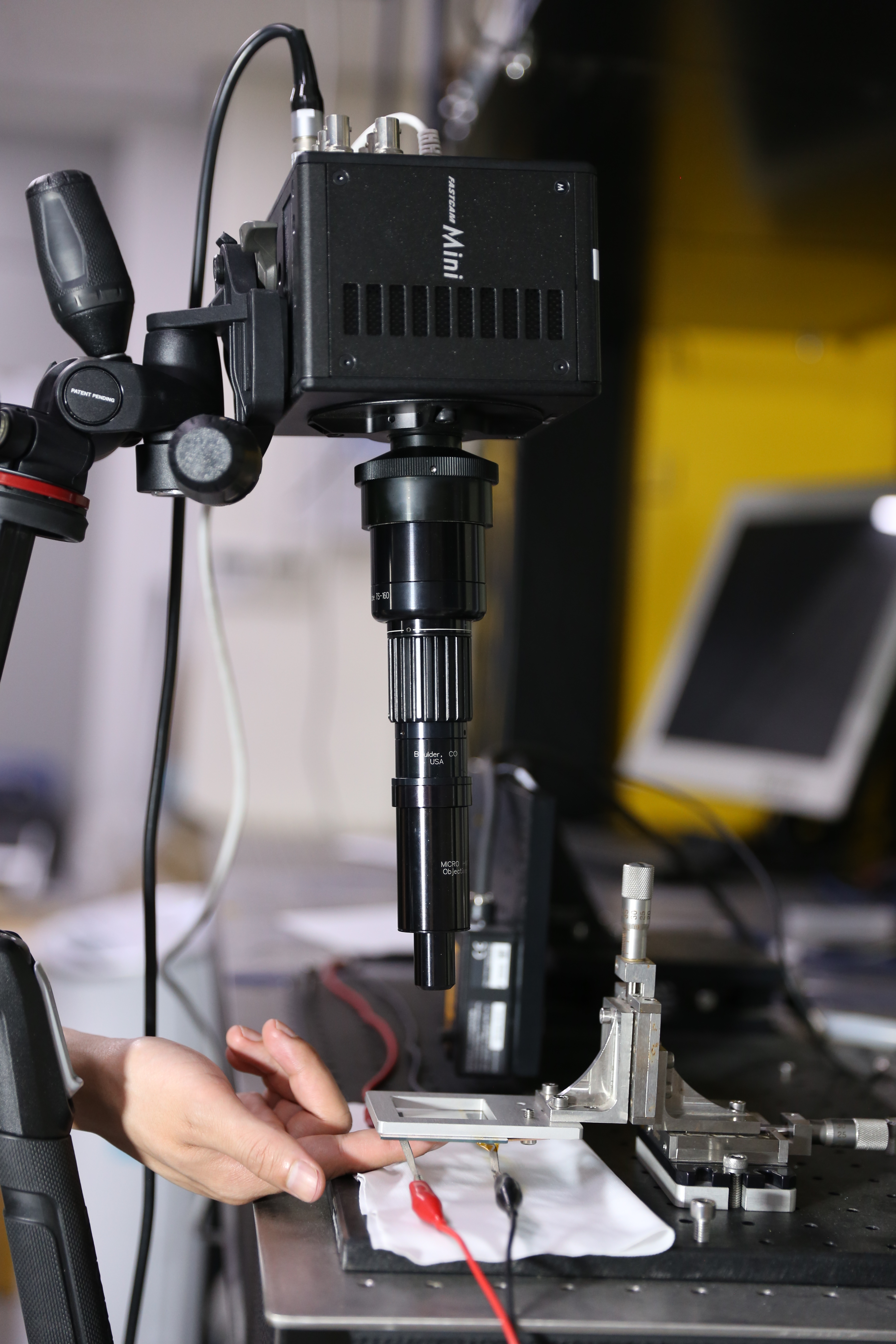


Figure S2. Experimental setup for observing the fretting vibration phenomenon.

**References**

1. De, S. *et al*. Silver Nanowire Networks as Flexible, Transparent, Conducting Films: Extremely High DC to Optical Conductivity Ratios. *ACS Nano* **3**, 1767-1774 (2009).

2. Liu, C. & Yu, X. Silver nanowire-based transparent, flexible, and conductive thin film. *Nanoscale Research Letters* **6**, 75 (2011).

3. Hu, L., Kim, H. S., Lee, J., Peumans, P. & Cui, Y. Scalable Coating and Properties of Transparent, Flexible, Silver Nanowire Electrodes. *ACS Nano* **4**, 2955-2963 (2010).

4. Choi, S. T., Kwon, J. O. & Bauer, F. Multilayered relaxor ferroelectric polymer actuators for low-voltage operation fabricated with an adhesion-mediated film transfer technique. *Sensors and Actuators A: Physical* **203**, 282 (2013).
